# Miniaturized chromatography-based lipidomics methods towards single-cell analysis

**DOI:** 10.1007/s00216-026-06488-0

**Published:** 2026-04-21

**Authors:** Klidel Fae Rellin, Michael Witting

**Affiliations:** 1 Metabolomics and Proteomics Core, Helmholtz Zentrum München German Research Center for Environmental Health, 85764 Neuherberg, Germany; 2 School of Chemistry, Aristotle University of Thessaloniki, Thessaloniki, Greece; 3 Chair of Analytical Food Chemistry, TUM School of Life Sciences, Technical University of Munich, Maximus-Von-Imhof-Forum 2, 85354 Freising-Weihenstephan, Germany

**Keywords:** Lipidomics, Mass spectrometry, Liquid chromatography, Ion mobility, NanoLC, Single cell lipidomics

## Abstract

Lipidomics provides detailed insight into lipid metabolism and cellular function, but conventional workflows typically rely on bulk samples that mask cellular heterogeneity. Advances in analytical chemistry are enabling lipid analysis from extremely limited material, driving the development of miniaturized chromatography-mass spectrometry (LC-MS) workflows for low-input and single-cell studies. This review summarizes recent progress in miniaturized chromatography-based lipidomics. Reducing chromatographic scale improves ionization efficiency, sensitivity, and separation performance while minimizing sample consumption. We discuss key enabling technologies, such as low-flow electrospray interfaces and the integration of ion mobility (IM) spectrometry as an orthogonal separation dimension for lipid identification. Methodological considerations for low-input lipidomics are also addressed, particularly sample preparation and quantitative challenges at picogram-scale analyte levels. Finally, we highlight future directions in automation, microfluidics, and multidimensional separations. Together, these developments position miniaturized chromatography as a critical platform for advancing single-cell lipidomics and high-resolution studies of lipid metabolism.

## Introduction

Lipidomics integrates advanced analytical chemistry approaches, particularly MS [1], to investigate lipid metabolism at a molecular level. The field aims to characterize and quantify lipids to elucidate, for example, nutrition- and disease-induced changes [2]. By annotating lipid classes and species and measuring their abundances, lipidomics generates detailed molecular profiles that reflect functional and metabolic states [2], including those shaped by diet, lifestyle, environment, or disease progression [3, 4]. This broad applicability arises from the structural diversity of lipids and their central roles in membrane architecture [5, 6], protein localization within macromolecular assemblies [5, 7], signaling [8, 9], and energy metabolism [5, 8].

The structural heterogeneity of lipids poses significant challenges for robust qualitative and quantitative analysis. Conventional MS-based lipidomics, which typically analyzes bulk tissues or cell populations, often masks cell-to-cell variability and limits mechanistic insights to microscale resolution. For example, cancer research often investigates tumor microenvironments, as some types of cancer are metabolically heterogeneous [10]. This would be difficult to model in cell culture, as cell-to-cell interactions can influence each other’s metabolic and nutrient demands [10]. Overcoming these limitations requires analytical strategies with exceptional sensitivity, structural resolution, and reproducibility. This gave rise to the field of single-cell analysis, such as single-cell transcriptomics, proteomics, and/or metabolomics/lipidomics.

Single-cell analysis relies on a wide range of analytical techniques to capture the heterogeneity that is often obscured in bulk measurements. MS-based techniques can be grouped into three complementary strategies: direct analysis via infusion or capillary-based sampling of isolated cells, imaging, and ultra-sensitive separations. Direct infusion or capillary-based sampling utilizes microprobe approaches where a single cell is aspirated or lysed and the extract is ionized with minimal upfront separation [11]. This maximizes throughput and minimizes sample losses, but it is vulnerable to ion suppression with limited isomer specificity. Mass spectrometry imaging (MSI), commonly done using matrix-assisted laser desorption/ionization (MALDI) and desorption electrospray ionization (DESI), is the go-to method for single-cell analysis as it provides spatial maps of biomolecules while retaining microenvironment information [12, 13]. MSI remains constrained by limited structural resolution [13], modest dynamic range [14], and challenges in absolute quantification [15]. These shortcomings highlight the need for complementary approaches capable of delivering high-coverage, quantitative lipid profiling at single-cell scale.

**Fig. 1:**
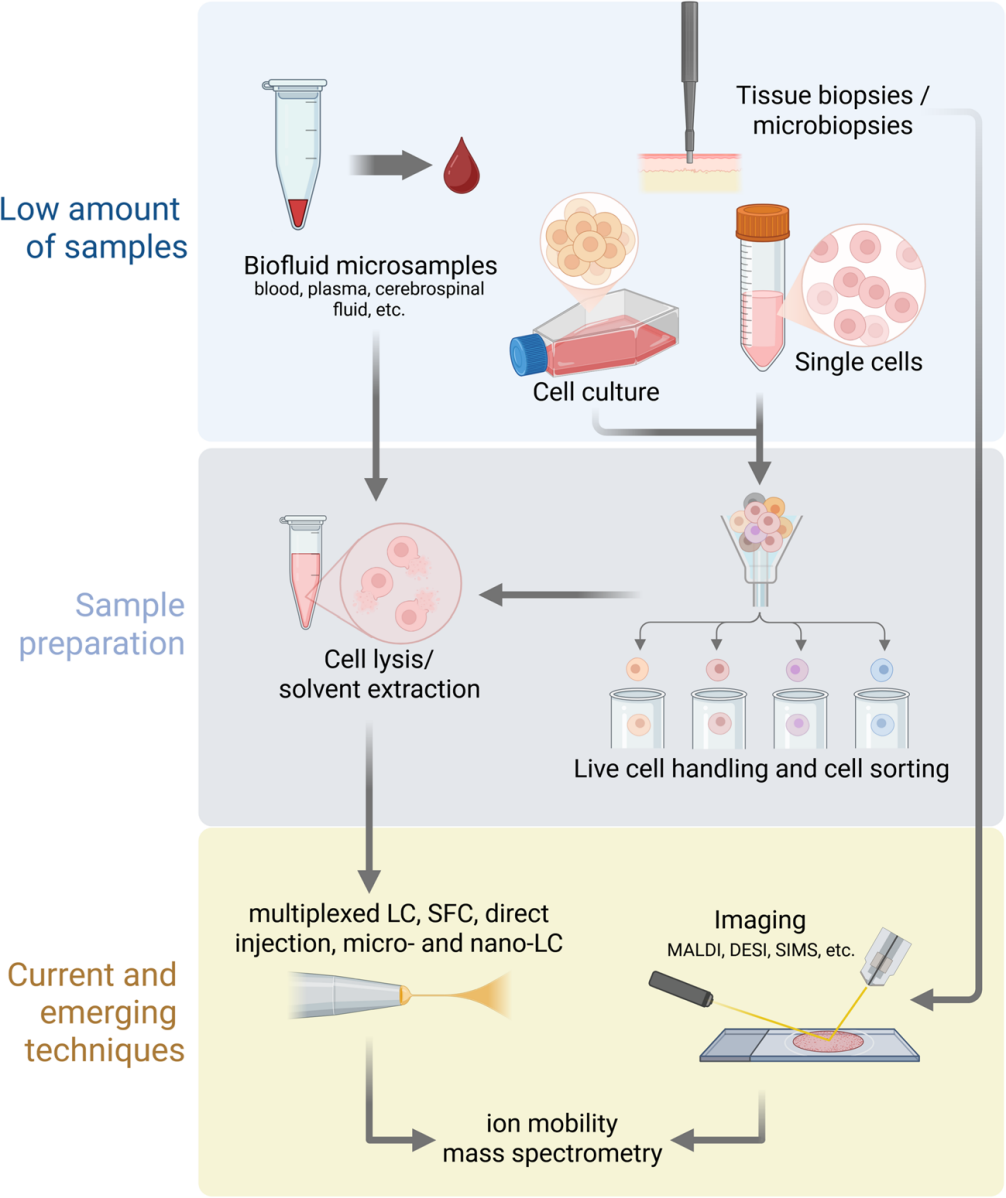
A succinct overview of the available workflows customarily used in analyzing low-volume/low amount of samples using MS

Chromatography-coupled MS has long been central to lipidomics, with workflows ranging from direct-infusion (“shotgun”) methods to liquid chromatography (LC), gas chromatography (GC), and supercritical fluid chromatography (SFC), the latter improving selectivity and mitigating ion suppression [16, 17]. Recent advances in high-resolution and tandem MS, including faster scan rates and enhanced sensitivity, together with modern data acquisition modes such as data-dependent (DDA), data-independent acquisition (DIA), and targeted monitoring strategies, have broadened lipid coverage, analytical throughput and accuracy of quantification. Building on these foundations, multiplexed LC-MS [18], IM-MS [19–27] and, most recently, miniaturized chromatography workflows, micro- and nanoflow LC [3, 28–34], have opened new avenues for high-sensitivity lipid analysis (Fig. 1).

Micro- and nanoflow LC coupled to advanced MS, sometimes in combination with IM separation, provides significant gains in sensitivity, sample economy, and separation power, particularly in resolving isomeric and isobaric lipids. These developments extend lipidomics to applications that demand ultra-low sample input, including single-cell analyses and large-scale perturbation studies. Unlike univariate readouts, lipidomics delivers multivariate molecular outputs, enabling simultaneous detection and quantification of hundreds to thousands of lipid species.

As with other omics fields, there is growing momentum toward single-cell resolution. In contrast to nucleic acids, which can be enzymatically amplified by polymerase chain reaction, proteins, metabolites, and lipids lack an equivalent technique, so their analysis is fundamentally mass-limited, making sensitivity, recovery, and ionization efficiency decisive for reliable measurements at ultra-low input. Single-cell proteomics, metabolomics, and lipidomics thus provide complementary functional readouts, with lipidomics offering unique insights into membrane biology, signaling pathways, and metabolic regulation.

In this review, we focus on innovations in miniaturized chromatography-based lipidomics, which falls on the third strategy: ultra-sensitive separations. In this context, *miniaturization* primarily refers to the scaling down of analytical systems, specifically chromatography, and their fluidics architecture. We discuss developments in microLC, nanoLC, and chip-based workflows; advances in low-flow interfaces and coupling strategies to conventional MS systems; the role of IM as an orthogonal separation dimension; and quantitative considerations at ultra-low sample input.

Equally critical to miniaturization is sample preparation, cleanup, and injection mechanisms that must be adapted to ultra-low analyte mass by minimizing transfers, adsorption, and hold-up volumes. Under these definitions, the “miniaturized workflows” we tackle in this paper are best viewed as scaled-down chromatography with low-loss sample handing coupled to conventional MS hardware. Meanwhile, data acquisition paradigms and downstream data analysis that are largely unchanged by physical scaling are not considered miniaturization and are discussed only insofar as they are direct consequences of low-input chromatography. We further highlight how these scaled-down approaches address current analytical challenges and position miniaturized chromatography as a cornerstone for the future of single-cell lipidomics in systems biology and disease research.

While the methodological choice in single-cell lipidomics is inherently dependent on the biological question, sample constraints, and experimental priorities, the analytical performance of each approach is governed by distinct operational boundaries. In this review, we do not prescribe specific workflows but instead highlight the characteristic trade-offs, performance limits, and failure modes associated with miniaturized chromatography-based lipidomics. By making these constraints explicit, we aim to provide a framework through which readers can interpret when and why these approaches succeed or become limiting, particularly in relation to complementary strategies such as MSI.

### From bulk to single-cell: why miniaturization matters in lipidomics

Classical lipidomics workflows were developed for bulk analyses of tissues, biofluids, microorganisms, or cultured cells, typically requiring microgram-to-milligram amounts of total lipid extracts [35]. While these approaches have been instrumental in mapping lipid metabolism and identifying disease-associated signatures, they inherently yield (cell or tissue) population-averaged data. Because biological systems are inherently heterogenous, this “bulk” perspective conceals cell-specific metabolic variations and obscures rare yet biologically significant phenotypes [36]. Lipid composition can differ substantially between neighboring cells due to differences in metabolic state, membrane turnover, or local microenvironment cues [37]. Capturing these subtle variations requires analytical platforms with sensitivity and precision orders of magnitude beyond those used in conventional workflows.

Miniaturization provides the analytical means to bridge this gap. By reducing the physical dimensions of chromatographic systems, fluidic interfaces, and ion sources, miniaturized workflows enable efficient handling of extremely small sample volumes while maintaining or even enhancing separation performance and ionization efficiency [38]. The transition to micro- and nanoLC significantly increases the concentration of analytes at the electrospray emitter; these lower flow rates generate smaller initial droplets, which accelerate solvent evaporation and improve ionization [39]. This mechanism effectively lowers detection limits and mitigates ion suppression [40]. Similarly, microfabricated devices and chip-based platforms minimize dead volume and sample loss, two major challenges when analyzing material from single or rare cell populations.

Within these miniaturized chromatography-based workflows, we treat single-cell as an upstream constraint in which cell isolation, nanoliter-scale extraction, and low-dead-volume injections are performed on an individual cell, and we distinguish this from single-cell-equivalent bulk dilutions that primarily benchmark instrumental sensitivity but may underrepresent pre-analytical loss and variance that dominate true single-cell input. However, these two methods are often intertwined in practice because one-cell equivalent bulk dilutions provide a controlled, scalable input required for method optimization while true single-cell measurements are essential in capturing the full error structure. Additionally, it remains to be determined, in a broad manner, how many single-cell measurements are required to achieve adequate statistical power, given that the variance contributions at ultra-low input arise from both true biological heterogeneity and workflow-imposed stochasticity.

These approaches therefore represent fundamentally different analytical regimes: true single-cell measurements preserve biological heterogeneity but are constrained by stochastic sampling and limited coverage, whereas single-cell-equivalent strategies improve analytical robustness while inherently averaging out cell-to-cell variability. We would like to point out that, while the first one (true single-cell measurement) is the ultimate goal, the usage of single-cell equivalents allows the development of analytical workflows and methods, such as chromatography, MS data acquisition, etc. Furthermore, single-cell equivalents are important as quality control samples, since pooled QCs are often not feasible due to low amounts of available true single-cell extracts.

Beyond improving sensitivity, miniaturization also facilitates a more faithful representation of the biological system. Reducing the required sample input allows the study of spatially restricted regions, such as microdissected tissue niches or organoid substructures, without pooling [41]. This aligns lipidomics with the broader spatial omics paradigm, where resolving biochemical heterogeneity is key to understanding tissue function and disease progression [42]. Finally, miniaturized workflows are inherently more resource-efficient, consuming significantly less solvent and generating less waste, thereby upholding the core principles of green analytical chemistry.

## Miniaturized chromatography platforms

Miniaturized LC workflows are defined by column internal diameters (ID) significantly smaller than those of conventional analytical LCs (2.1–4.6 mm), typically operating at flow rates in the µL to nL per minute range [43]. By reducing the column diameter, these systems minimize on-column dilution, thereby increasing analyte concentration at the detector and maximizing ionization efficiency with ultra-fine electrospray droplets [39]. The term *miniaturized* thus encompasses microLC, nanoLC, and capillary LC formats, as well as integrated chip-based devices designed to reduce dead volume and improve reproducibility [44]. Collectively, these workflows represent a shift from traditional, bulk-oriented separations toward highly efficient, resource-conserving platforms that enable comprehensive lipid profiling from scarce biological material. Table 1 summarizes these formats. Rather than representing a linear improvement hierarchy, these formats define distinct operating regimes in which gains in sensitivity, robustness, or throughput are achieved at the expense of the others.

**Table 1:** Summary of miniaturized LC methods

Format	Typical column ID	Typical flow rate	Usual injection volume	Sensitivity (relative)	Robustness	Throughput	Key pros	Key cons	Best-fit applications
MicroLC	0.3–1.0 mm	1–50 µL·min⁻^1^	0.5–5 µL	moderate	high	high	Good compromise of sensitivity and robustness; easier plumbing; compatible with most ESI sources	More solvent use than nano/capillary; less sensitive than nanoLC	Low-input cohorts, small biopsies, medium-throughput studies
NanoLC	50–100 µm	0.1–0.5 µL·min⁻^1^ (100–500 nL·min⁻^1^)	20–500 nL	high	moderate	low-to-moderate	Highest ionization efficiency; best for scarce material; reduced ion suppression	More delicate fluidics; emitter stability critical; longer setup/maintenance	Single-cell/rare cells; deep coverage; discovery profiling
Capillary LC	50–300 µm	0.5–10 µL·min⁻^1^	100 nL–2 µL	moderate	moderate-to-high	moderate-to-high	Bridges nano and analytical scales; flexible phases/geometries; easier operation than nano	Sensitivity below nanoLC; still requires careful dead-volume control	Routine low-input work; class-oriented separations; method development
Chip-based LC	10–150 µm (on-chip channels)	0.05–2 µL·min⁻^1^	10–500 nL	moderate-to-high	moderate-to-high	high	Minimal dead volume; integrated emitter; excellent reproducibility; amenable to automation/multiplexing	Device availability/cost; fewer stationary-phase options; thermal/pressure limits	High-throughput low-input workflows; standardized clinical/translational pipelines

### Micro- and nanoflow LC

LC miniaturization will serve as one of the primary engines driving the transition from bulk-tissue lipidomics to single-cell resolution. By scaling down column IDs, these platforms mitigate the radial dilution effect that plagues analytical flow. MicroLC (1–100 µL/min) and nanoLC (0.1–0.5 µL/min, Table 1) facilitate more efficient desolvation and a higher percentage of analyte transfer into the gas phase, resulting in superior mass sensitivity and the detection of low-abundance signaling lipids that are otherwise lost in the baseline noise [45].

NanoLC could also potentially emerge as a standard for single-cell lipidomics because it maximizes analyte utilization and minimizes matrix-induced ion suppression through ultra-low flow rates, while simultaneously providing high peak capacity for complex lipidome separation. However, the “sensitivity and throughput tax” for nanoLC is technical complexity: it requires meticulous control over fluidic connections and specialized emitter designs to prevent clogging and to ensure a stable spray [46]. Conversely, as demonstrated in proteomics applications, nanoLC offers significantly higher throughput and operational robustness at a modest cost to sensitivity. This makes it particularly well-suited for clinical cohorts involving rare cell populations or needle biopsies, where the sample is limited but the need for reproducible, large-scale data is paramount [47].

These advantages show a clear operational boundary: nanoLC performance becomes increasingly fragile in complex matrices and across prolonged acquisition sequences, where emitter instability, clogging, and matrix-derived residue effects can dominate variance and reduce effective throughput despite high theoretical sensitivity. Coming back to a point previously raised: Single-cell equivalent measurements can help to optimize new designs and methods in order to improve workflows for true single-cell applications.

### Capillary and chip-based LC

Capillary LC, utilizing columns with ID typically ranging from 50 to 300 µm, serves as a critical intermediary between the high-sensitivity, yet often temperamental, nanoLC (< 100 µm ID) and the high-throughput, solvent-intensive analytical LC (< 4.6 mm ID). By operating at flow rates of 0.5–10 µL/min, capillary formats mitigate extra-column dispersion issues associated with smaller column diameters while offering substantially higher pressure tolerance and mechanical robustness than nanoLC and nano-electrospray (nanoESI) setups [48, 49].

Recent advancements have led to the emergence of integrated microfluidic LC platforms, commonly known as *lab-on-a-chip* systems. These devices integrate the analytical separation channel, solid-phase extraction (SPE) enrichment traps, and ESI nozzle/emitter onto a single microfabricated substrate, typically made from polyimide, glass, or silicon-based materials. This high level of integration enables precise control of fluid handling while minimizing dead volumes and system complexity [50]. By eliminating conventional fittings and tubing required to perform LC-MS, lab-on-a-chip platforms substantially preserve peak capacity and shape, and retention time reproducibility [50]. Moreover, sample loss is drastically reduced with on-chip integration, and together with nanoESI interfaces, it enables robust analysis at picogram to femtogram levels [51].

While initially developed for proteomics, micropillar array columns (µPAC) can also be implemented in chip-based formats. These microfabricated silicon columns consist of perfectly ordered stationary-phase structures, offering exceptional permeability, minimal eddy diffusion, and near-theoretical separation efficiency. As a result, µPAC can outperform traditional packed nanoLC columns for low-input analysis [52, 53]. Consequently, capillary and chip-based LCs occupy a transitional regime in which gains in robustness are achieved at the cost of reduced sensitivity relative to nanoLC, making it less suitable for extremely low-input applications where ion statistics become limiting. However, the increasing sensitivity of modern MS instruments potentially will cause a shift from nanoLC towards higher-flow rate, but still miniaturized chromatographic setups, even in proteomics.

## Coupling miniaturized chromatography to MS

The analytical advantages of miniaturized chromatography can only be fully realized when effectively coupled to high-performance MS via robust, efficient interfacing strategies. As column diameters and flow rates decrease, efficient ion transfer and stable ionization become critical to preserve sensitivity and reproducibility. Consequently, the interface between the chromatographic outlet and the MS is a central element of miniaturized LC-MS workflows, governing not only signal stability but also compatibility with multidimensional separation techniques such as IM.

Improvements in emitter design, ionization interfaces, and microfluidics have enhanced the performance of miniaturized LC-MS. In parallel, the development of compact, high-resolution IM modules has further expanded their analytical scope.

### Low-flow electrospray interfaces and emitter designs

**Fig. 2:**
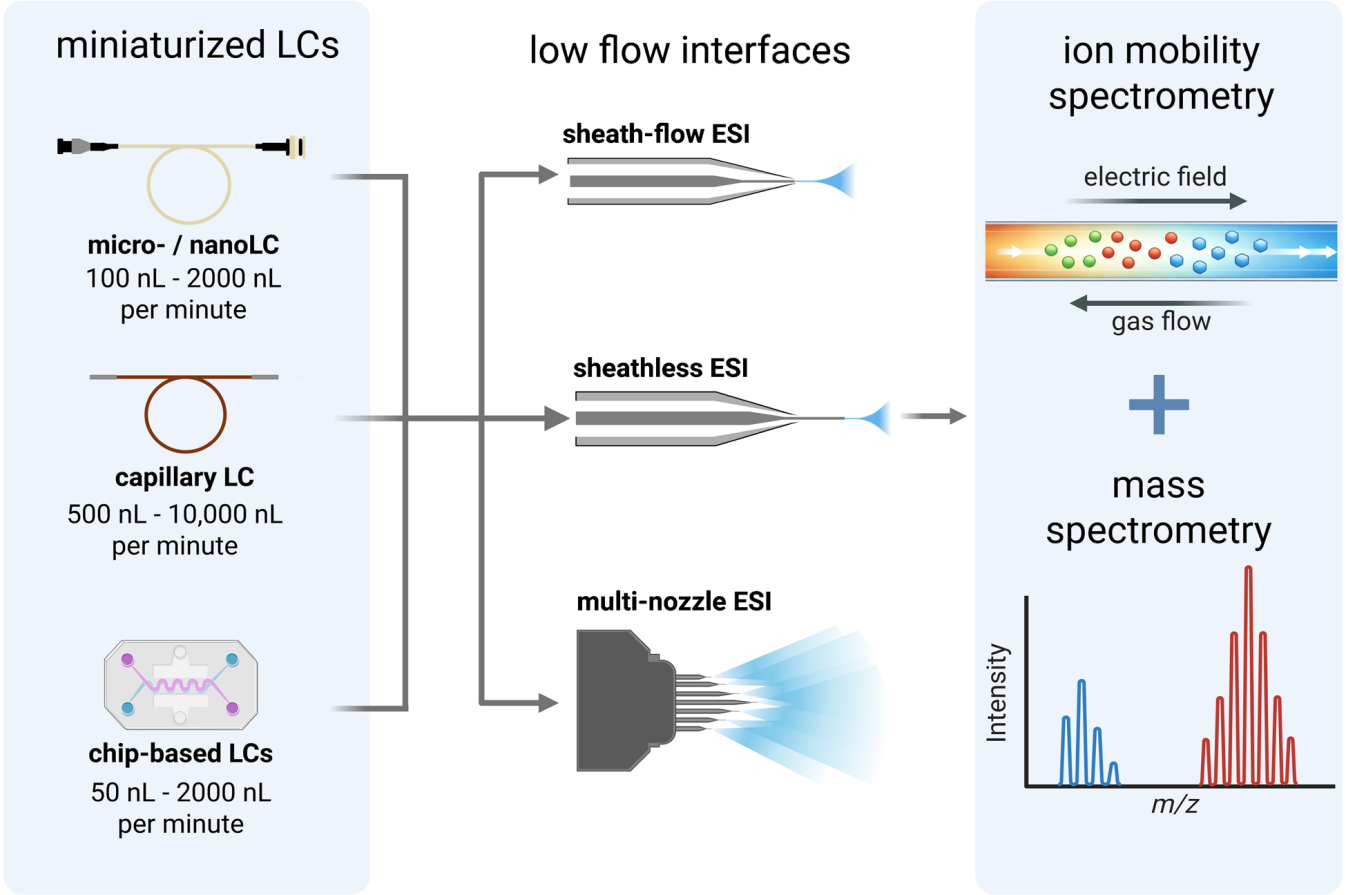
Schematic diagram illustrating the integration of miniaturized LC-MS and IMS. Three LC formats are depicted on the left—micro/nanoLC, capillary LC, and chip-based LC—each operating at low nanoliter-per-minute flow rates to enable high sensitivity and reduced sample consumption

The performance of miniaturized chromatography workflows is intrinsically linked to the efficiency, stability, and reproducibility of ESI. At reduced flow rates, typically in the range of 0.1–10 µL/min, the generation of smaller initial droplet sizes leads to enhanced ionization efficiency and diminished ion suppression from co-eluting matrix components [39, 54]. This enhancement is especially beneficial to nanoLC-MS, where analyte concentrations are inherently low. Stable operation at these flow regimes also requires precise fluidic control and robust emitter geometries (Fig. 2). Modern nanoLC systems incorporate integrated spray tips or metal-coated capillaries to prevent dead volume issues and ensure electrical stability during long acquisitions [55]. Conventional sheath-flow emitters, which introduce an auxiliary liquid around the analyte stream, provide mechanical stability but further dilute the analyte. In contrast, sheathless designs eliminate the auxiliary flow, delivering the entire effluent directly to the MS inlet.

The nanoESI emitter itself is often the dominant determinant of stability and reproducibility. Most emitters are fabricated from pulled or chemically etched glass or fused silica, in which taper angle, tip ID, and end-face morphology govern the electric field strength and the accessible cone-jet regime. Smaller orifice diameters generally lower the flow rate required for stable emission and can increase sensitivity, but they also raise the risk of clogging and variability in spray onset voltage [56]. Conductive coatings or internal electrodes are commonly used to provide reliable electrical contact without excessive electrochemical reactions in the sample. Importantly, the relationship between emitter ID, surface wettability, and solution conductivity sets the operating window for stable Taylor cone formation, and departures from these values can lead to pulsating or multi-jet modes that degrade quantitative performance [57].

Emitter geometry has also evolved from simple single-barrel tips to structured formats that enable new functions. Multibarrel and theta-glass emitters permit rapid on-tip mixing for reaction monitoring, hydrogen-deuterium exchange, or supercharging strategies, effectively turning the emitter into a microreactor while maintaining nanoESI conditions [58].

Multinozzle (multi-emitter) ESI sources extend these ideas by parallelizing the spray. Instead of a single Taylor cone, arrays of nozzles generate multiple cones and plumes, increasing the total ion current at a given per-nozzle flow while preserving the small droplet sizes characteristic of low-flow ESI. This can improve sensitivity and dynamic range without resorting to higher single-emitter flow rates that would otherwise enlarge droplets and reduce ionization efficiency. Early microfabricated multinozzle emitters demonstrated higher signal and improved tolerance to higher total flow through parallelization [59]. Later work with emitter arrays and multiplexed nanoESI sources showed gains in throughput and robustness. However, careful control of nozzle-to-nozzle uniformity, electric-field screening between adjacent sprays, and plume-inlet alignment is essential to realize theoretical benefits [56, 60]. A demonstration of the adaptability of multinozzle ESI with low-flow LC is shown in a paper by Girel et al. [61]. In the reported configuration, a ~ 3 μL/min microflow separation is coupled to a microfabricated multinozzle electrospray chip featuring five nozzles, each operating at ~ 600 nL/min, effectively generating parallel nanoelectrosprays from a single LC run. This design increases the total electrospray surface area and electric field density, producing smaller, highly charged droplets that enhance desolvation and ion formation while reducing matrix effects. As a result, the system delivers markedly higher sensitivity, showing a ~ 16-fold median signal increase across deuterated lipid standards, along with improved control of adduct formation and milder in-source fragmentation [61].

### Integration with ion mobility spectrometry

Coupling low-flow LC to IM-MS is attractive because the sensitivity benefits of low-flow ESI (small droplets, efficient desolvation, reduced dilution) compound with the orthogonal gas-phase separation of IM, which reduces spectral congestion and improves selectivity for co-eluting species. Practically, successful integration hinges on managing the mismatch between LC’s narrow, fast chromatographic peaks at low flow and the ion-optics/IM duty cycle: interfaces must preserve peak sharpness (low dead volume), maintain stable nanoESI at 50–300 nL/min, and deliver high ion transmission into the IM cell despite low total spray currents. Early ESI-IM implementations already recognized the need for ion accumulation/trapping upstream of the drift region to improve duty cycle and sensitivity when feeding IM with continuous ESI beams [62].

Modern IM platforms illustrate how this has matured. Trapped ion mobility spectrometry (TIMS) is particularly compatible with LC because ions can be accumulated and then released in mobility order, and in timsTOF instruments, this is paired with parallel accumulation-serial fragmentation (PASEF) to improve sequencing speed and sensitivity—features that have been explicitly discussed in TIMS-PASEF reviews focused on LC-IM-MS workflows [63]. In practice, many high-sensitivity TIMS studies, especially in proteomics, continue to rely on nanoLC because it maximizes ionization efficiency per analyte amount; for example, nanoLC-TIMS lipidomics demonstrated high sensitivity while leveraging IM separation to address isomers and co-eluting lipids [21]. At the same time, there is growing interest in carefully optimized nano- and microflow regimes to balance robustness and throughput with IM-MS performance, driven by developments in pumps/columns and ion transfer designs that maintain high ion utilization even as flow is increased beyond classic nanoLC [64, 65].

FAIMS (high-field asymmetric waveform ion mobility) adds another integration pattern: it is typically placed between the ESI source and the MS inlet and acts as an ion filter that can reduce chemical noise and improve analysis depth, but it also introduces additional ion losses and therefore benefits strongly from efficient low-flow ionization and careful source-inlet alignment. Reviews on FAIMS emphasize the centrality of ion transmission and focusing (e.g., funneling) through the FAIMS region and into the MS, which becomes even more consequential at low-flow ion currents [66, 67]. In single-cell and other mass-limited proteomics, the combination of ultra-low-flow nanoLC with FAIMS-enabled separations has been used to boost identifications by reducing background and disentangling co-isolated precursors [68].

Finally, emerging “ion-utilization” architectures seek to ensure that low-flow LC’s limited ion current is not squandered before, during, or after mobility separation. Structures for Lossless Ion Manipulations (SLIM) exemplify this direction. SLIM-based IM modules emphasize high transmission and the ability to accumulate/store IM-selected ions, which is conceptually well matched to low-flow LC, where maximizing usable ions per injected amount is paramount [69]. Across TIMS, FAIMS, drift-tube IM, and SLIM, the key engineering theme is the same: co-optimize (1) low-dead-volume LC-ESI plumbing, (2) stable low-flow emitters and source fields, and (3) ion trapping/funneling and timing strategies that reconcile LC peak widths with IM scan/acquisition cadence to preserve both chromatographic and mobility peak capacity while maintaining high sensitivity [55].

While IMS can add a quantifiable orthogonal dimension to LC-MS lipidomics, its best benefit at the single-cell scale is providing more chemical information from restricted samples. In terms of performance metrics, modern high-resolution IMS platforms can reach substantially higher resolving power than earlier time-dispersive IMS implementations. TIMS, for example, has a reported resolving power of up to 400 under optimized conditions, albeit with the familiar tradeoffs on throughput [70]. In practical terms, the analytical gain from ion mobility in lipidomics is best understood as a conditional increase in separation capacity rather than a universal resolution solution. When combined with LC and MS/MS, IM can reduce spectral congestion and improve feature annotation, particularly for co-eluting lipid classes and select isomeric and isobaric species. However, many lipid isomers exhibit only small differences in collision cross section, often below the resolving capability of current IM platforms, limiting their standalone discriminative power. As a result, the primary benefit of IM in miniaturized LC-MS workflows is improved confidence in annotation through multidimensional separation, rather than complete structural resolution.

In lipidomics, ion neutral collision cross section (CCS) functions as an additional molecular descriptor that improves feature filtering and can separate select isomeric families when combined with LC and MS/MS. Foundational LC-IMS-MS studies demonstrate improved discrimination of biologically relevant lipid isomers in complex matrices, but also illustrate that CCS differences between many lipid isomers are subtle and frequently require multi-dimensional evidence rather than CCS alone [71]. CCS measurements can be highly reproducible across laboratories and instruments when standardized methodologies are used, which supports CCS as a robust constraint for annotation [72]. However, CCS database coverage remains incomplete and uneven across lipid classes and adduct types, motivating cautious use of CCS trendlines, explicit uncertainty windows, and false-positive control rather than treating CCS as a unique identifier [73–75]. Machine-learning CCS prediction frameworks such as LipidCCS can expand coverage and enable plausibility checks, but predicted CCS values should be treated as supportive priors and validated with experimental CCS and MS/MS wherever possible [75]. These observations indicate that IM should not be interpreted as a universal solution for isomer/isobar resolution, but rather as a conditional separation dimension whose effectiveness depends on the physicochemical diversity of the analytes and its integration with chromatographic and MS/MS information.

Finally, the interaction between IMS and acquisition mode is non-trivial: while mobility separation can reduce spectral congestion, DIA implementations (for example, DIA-PASEF) can still suffer ambiguous fragment-to-precursor assignment when lipids share similar retention time and mobility, which is amplified under low-ion-count single-cell conditions [76]. Practically, IMS improves confidence most when integrated into a multidimensional identification framework (RT, MS/MS fragmentation rules, CCS windows), rather than being presented as a standalone solution for isomer resolution or DIA deconvolution [77]. Hence, IM should be viewed as an enabling but non-sufficient dimension, whose analytical value emerges primarily in combination with other separation techniques.

## Less is more: sample preparation for low-input lipidomics

Sample preparation represents one of the most critical and technically demanding stages in miniaturized and single-cell lipidomics. At such scales, every step—from cell isolation and lysis to solvent extraction and transfer—can drastically affect analytical recovery, reproducibility, and quantitative accuracy. Unlike conventional bulk lipidomics, where sample size can compensate for handling inefficiencies, low-input workflows operate near the detection limits of current instrumentation. Therefore, maximizing extraction efficiency, minimizing sample loss, and ensuring chemical compatibility with downstream nanoLC-MS are central to method design. Recent developments have introduced a range of strategies, including solvent-miniaturized extractions, microfluidic automation, and integrated nanospray sampling systems, all aimed at preserving analyte integrity while reducing experimental variability.

### Extraction protocols for limited samples

Lipidomics at limited input (µL-scale biofluids, 10^3^–10^5^ cells, sub-mg tissues) demands protocols that maximize recovery across lipid classes while minimizing handling losses, dilution, and artifactual oxidation/hydrolysis. Most workflows still rely on biphasic liquid-liquid extraction (LLE) chemistries, but with microscale adaptations that preserve solvent ratios while reducing absolute volumes and transfers. The classic Folch and Bligh-Dyer systems remain foundational for broad lipid coverage and efficient protein precipitation, and when miniaturized, maintaining the original phase ratios is key to reproducible partitioning [78, 79]. For small volumes, many laboratories favor MTBE-based extraction, which places the organic phase on top and delivers strong performance for plasma and small tissues [80]. BUME (butanol/methanol) is another chloroform-free option compatible with small volumes and automation, with good coverage of neutral and polar lipids [81].

At low inputs, single-vessel strategies are especially valuable: perform lysis, extraction, and reconstitution in the same low-bind vial; avoid multiple dry-down/reconstitution cycles; and add class-representative internal standards before extraction to track recovery and normalize variability. Community best practices emphasize strict control of solvent purity, temperature, and antioxidants to limit oxidation, as these are critical when absolute lipid amounts are small and artifactual changes can rival biological effects.

Cleanup steps in lipidomics can reduce salts and proteins that contribute to ion suppression, improve chromatographic stability, and enable class-specific enrichment, but, in the context of limited sample amounts, they must be applied judiciously because every additional manipulation introduces the risk of analyte loss and variability. Consequently, cleanup for low-input lipidomics is best kept purpose-driven. Minimal SPE, implemented in cartridges or 96-well plates, is widely used for desalting and class-based fractionation (e.g., aminopropyl or silica schemes for neutral versus polar lipids) because it offers relatively reproducible separations, microscale scalability, and compatibility with automation. However, for small samples, it is critical to precondition sorbents with minimal volumes, use small elution volumes compatible with LC starting conditions, and include internal standards in each fraction to monitor recovery.

Hybrid protein-precipitation or filtration plates provide a low-handling option for plasma or serum extracts by removing precipitated proteins and particulates before LC-MS. However, they function primarily as matrix cleanup tools rather than true lipid class fractionation. Gel-permeation or size-exclusion approaches can remove polymers and very large interferences. Still, their tendency to dilute samples and introduce adsorptive losses makes them less suitable for routine low-amount work and more appropriate for targeted contamination problems. These considerations align with community recommendations that emphasize standardization, recovery tracking, and minimizing unnecessary steps in MS-based lipidomics workflows [82].

### Microfluidic and automated handling approaches

Microfluidic handling and automated sampling have become central to single-cell lipidomics because they address the extreme constraints of volume, sensitivity, and contamination that arise when measuring lipids from individual cells. Lipids are chemically heterogeneous, spanning wide ranges of polarity and abundance. They are highly prone to surface adsorption and rapid metabolic turnover, making conventional bulk or plate-based workflows poorly suited for true single-cell resolution. Microfluidic systems, by contrast, operate at nano- to picoliter scales with tightly controlled fluid dynamics, enabling rapid isolation, lysis, and extraction while minimizing dilution and analyte loss. These features align well with the broader trajectory of single-cell metabolomics, where the importance of miniaturized, low-loss handling has been emphasized for over a decade [36].

A major advantage of microfluidics in single-cell lipidomics is its ability to provide deterministic, gentle cell isolation. In one study [83], an automated, microfluidics-based workflow for single-cell lipidomics was developed that integrates live-cell isolation with conventional LC-MS, aiming to make single-cell lipid profiling more accessible and higher throughput. The authors optimized a commercial microfluidic single-cell picking platform to reduce lipid contamination and ionization suppression, identifying key sources of background signal, including fetal bovine serum, albumin coatings, and carrier buffers. By switching media, diluting human-derived albumin, and carefully evaluating buffer composition, they substantially reduced blank contamination and increased lipid detection, achieving ~ 200 lipid features per single cell—comparable to established capillary sampling methods. A direct comparison showed that microfluidic and capillary sampling yield similar lipid profiles and coverage, but microfluidics offers lower variability, automation, and ~ 80% faster throughput, albeit without spatial information [83].

Building on advances in automated and contamination-aware single-cell lipidomics, recent work extends automation upstream to intelligent single-cell manipulation. Jia et al*.* present an active-matrix digital microfluidics (AM-DMF) platform that integrates computer vision and large language models (LLMs) to fully automate droplet generation, cell recognition, sorting, and routing [84]. A three-class detection model distinguishes droplets, cells, and oil bubbles to reduce misclassification, while a droplet-movement strategy helps reveal cells hidden at droplet edges. In parallel, a Llama-3-based droplet path generation model converts user prompts into movement and splitting paths, eliminating manual design. Together, these innovations achieve >98% cell identification precision, a single-cell generation rate above 25%, and high-throughput droplet handling, positioning AI-enabled digital microfluidics as a scalable, intelligent front end for downstream single-cell omics workflows [84].

## Throughput tax: the uptime costs of miniaturized LC-MS

Miniaturized LC-MS taxes throughput less through runtime alone and more through uptime erosion, and sample processing. MicroLC is often positioned as a pragmatic throughput regime because it retains chromatographic separation while improving robustness and sustained sample load capacity. A clear example is the high-throughput microbore LC-MS lipidomics workflow reported by Gadara, et al*.* [85]*,* which explicitly targets throughput and quantitative stability for routine profiling. MicroLC can also be paired with specialized ion-source engineering to increase ionization efficiency at higher flow than nanoLC, as demonstrated by Girel et al*.* [61].

Capillary LC occupies an intermediate space where separation performance and ionization efficiency can be improved while keeping plumbing demands below the most failure-prone nanoflow extremes. Capillary LC methods have been used to increase lipid identification through a combination of reduced flow, nanoESI, and optimized MS/MS acquisition as demonstrated by He et al. [86] (Table 2)*.* Capillary ultrahigh-pressure LC has also been applied for lipid separations to increase peak capacity and isomer resolution at high backpressure [87].

**Table 2:** Summary of throughput and constraints associated with miniaturized LC-MS lipidomics workflows in single-cell applications

Format	Example implementation(s) in single-cell lipidomics	Reported LC method	Theoretical max injections per day (24 h)	Throughput Tax (dominant single-cell constraint)
NanoLC-MS	Patch clamp-assisted single-neuron lipidomics using optimized nanoLC-MS [88]	~ 51 min (gradient + 10 min salt wash + re-equilibration)	~ 28/day	Highest sensitivity leverage, but uptime is fragile: emitter fouling and clogging (explicitly motivates extended salt wash), spray intermittency, low tolerance to particulates and salts, and high penalty per failed run because each cell is irrecoverable
Capillary LC-MS	Capillary LC + nanoESI approach used to increase lipid IDs in a single LC–MS run (bulk plasma lipidomics; method principles translate directly to low-input) [86]	60 min	24/day	Intermediate regime: improved robustness vs strict nanoLC, but still sensitive to dead-volume and adsorption at low input; gradient times often remain long for coverage, which limits true single-cell throughput unless aggressively shortened
MicroLC-MS	Single-cell untargeted lipidomics using microflow LC–MS/MS with 15 min LC gradient and live-cell sampling coupling [89]	15 min	96/day	Lower uptime tax: higher tolerance to minor leaks and particulates, more stable spray, faster re-equilibration. Main constraint becomes maintaining MS^2^ coverage and ID confidence at single-cell ion budgets, plus QC load and carryover control over long batches
Chip-based LC	Microfluidic nano-HPLC-Chip integrates enrichment + analytical column + nanospray on-chip to remove fittings and improve nanospray stability (demonstrated for lipid separations, including polar lipids like gangliosides; conceptually relevant for low-input) [90]	Example chip method includes a long gradient segment (reported as “40 min runs” with < 0.3 min RT variation; peak areas < 1% RSD reported)	~ 36/day if 40 min	Reduced plumbing complexity (fewer dead-volume junctions) and often better day-to-day reproducibility, but constraints shift to chip lifetime/cost, limited stationary-phase flexibility, and still nanoflow-like vulnerability to matrix deposition at the emitter

NanoLC provides the strongest concentration sensitivity leverage, but it is also the regime where the throughput tax is the highest because the system becomes extremely sensitive to dead volume, emitter condition, clogging, spray intermittency, and matrix-derived residue effects. Multiple lipidomics studies explicitly develop nanoLC workflows to exploit sensitivity while acknowledging the technical burden. Danne-Rasche et al. established a reproducible nanoLC-nanoESI strategy and reported gains in lipid identification and measurement performance relative to higher-flow approaches [29]. Buzatto et al*.* developed a nanoLC workflow for high-sensitivity global lipidomics [34], explicitly targeting small sample amounts where robustness and maintenance become part of method reality. Opti-nQL [30] is another example of a nanoLC lipidomics method designed for sensitivity and reproducibility across biological matrices, reflecting how nanoflow lipidomics often requires workflow-level standardization to keep uptime losses from dominating output.

Across these scales, throughput in miniaturized lipidomics is best defined not by gradient time, but by the number of successful, QC-compliant injections per unit time under realistic conditions. In practice, this makes throughput a function of system robustness, where emitter stability, clogging, and failure rates are accounted for, rather than runtime alone. Micro- and capillary LC therefore maximize usable throughput for cohort studies, while nanoLC maximizes depth per injection for scarce samples, subject to controlling downtime and reruns. A current major goal of analytical developments for single-cell lipidomics is to improve this robustness for true single-cell applications.

### Quantification challenges at single-cell scale

(Absolute) quantification in miniaturized chromatography-based lipidomics becomes difficult not because LC-MS cannot be quantitative, but because variance terms that are negligible at bulk scale dominate at picogram to nanogram lipid mass. While quantification can be often achieved at single-cell equivalent levels, true single-cell applications require new approaches overcoming several bottlenecks.

The first bottleneck is internal standard strategy: for robust class-wise and species-wise quantification, isotope-labeled standards should ideally be added as early as possible (pre-extraction) to track recovery and matrix effects, yet at single-cell volumes the act of dosing standards itself can introduce variability unless dispensing is tightly controlled and adsorption is minimized. Community guidance stresses early IS addition and transparent reporting of IS composition and placement but also highlights that imperfect IS coverage across lipid classes and acyl-chain diversity limits accuracy when class heterogeneity is high. In order to achieve dosing of IS, systems capable of nL dispensing are required, which are often not available at standard lipidomics laboratories. Low amounts of solvent containing IS can be added to collection tubes for single-cells, but often high evaporation rates of these solvents cause more problems and heterogeneity than they solve.

A second constraint is ion suppression and response non-linearity: even with chromatography, co-elution within and across lipid classes produces time-varying competition during ESI, which becomes particularly punitive when analyte ion counts are sparse and the measurement is effectively ion-count limited. This is one reason why nanoESI and low-flow regimes can improve tolerance to suppression, yet they do not eliminate suppression driven by unresolved co-elution and matrix residues. These constraints arise from fundamental physical limitations, including random ion sampling and matrix-dependent ionization efficiency, which place an upper bound on the extent to which absolute quantification can be achieved at true single-cell resolution.

Furthermore, almost all miniaturized systems use RP-based separation. Therefore, the IS might only co-elute with some lipids of the same lipid class, especially if long separation methods as used in nanoLC are used. However, coelution of IS with the target analyte is a prerequisite for accurate absolute quantification of lipids. Alternative IS sources, such as extracts from isotopically labeled cells potentially solve this problem as each lipid would have its own internal standard for normalization and quantification.

Single-cell workflows also face recovery and carryover artifacts that are often underappreciated in “single-cell-equivalent” dilution benchmarks. At high surface-area-to-volume ratios, adsorption to plastics, vial inserts, emitter surfaces, and dead volumes can create class-dependent losses and memory effects; these losses are random and can masquerade as biological heterogeneity if they are not tracked with process controls and QC criteria. Best-practice guidelines emphasize reporting extraction conditions, injection hardware, carryover checks, and QC schemes because these factors directly shape quantitative fidelity in lipidomics (Table 3).

**Table 3:** Quantitative benchmarks from representative lipidomics papers utilizing miniaturized LC regimes

	MicroLC-MS	Capillary LC-MS	NanoLC-MS
Representative lipidomics papers	Gadara et al. [85]	He et al*.* [86]	Cattaneo et al*. *[30]
Lipid annotation basis as implemented in the study	Species-level quantification (351 lipids quantified in their reported workflow)	Lipid identifications filtered for adducts, dimers, in-source fragments, isotopes, and RT mismatch; relative quantification with injection replicates (RSD calculated)	Semi-targeted IDs via LipidView; additional untargeted identification via SMFinder with database matching using exact mass and MS/MS, with and without FDR filtering (multiple confidence levels)
Lipid IDs	351 lipid species quantified in a 15 min LC–MS analysis; robustness shown over 303 injections (~ 75 h)	Over 1,500 lipids identified and relatively quantified within a 60 min capillary LC-MS analysis	In nanoflow, 608 (iPSC) and 698 (MEF) total lipid species identified; 451–559 lipids per run (average identified and quantified per run depending on sample)
Reproducibility metric	Median CV 12.95% across the 351 lipid species; 26 lipids had CV > 30%	Median CV 0.014% across replicate injections	Authors report high stability and highly reproducible peak areas and RT for internal standards across 0–75 days, and state nano-flow decreases CV relative to micro-flow
Peak capacity/separation proxy	Not reported	FWHM 4–9 s for isotope-labeled internal standards under 60 min conditions (used here as a peak-width proxy; calculated)	Not reported

Finally, absolute quantification is rarely straightforward at true single-cell input: without validated recovery for each lipid class, matched isotope standards, and calibration approaches compatible with nanoliter extraction volumes, most studies are realistically constrained to relative quantification (within-run or batch-corrected) or semi-absolute class estimates. Recent LC-MS single-cell lipidomics implementations underscore this point by focusing on method validation, reproducibility controls, and careful interpretation of intensities rather than claiming universal absolute concentrations across the lipidome (Table 4).

**Table 4:** Quantitative benchmarks for true single-cell versus one-cell equivalent miniaturized LC-MS lipidomics from representative studies

Study	Regime	Sampling unit	LC scale, runtime	Annotation basis	Lipid/cell	Bulk dilution	Reproducibility metric
Merrill et al*. *[88]	True single-cell	Patch clamp-assisted capture of identified hippocampal neurons	NanoLC	IDs based mainly on accurate m/z + RT matching to highly diluted tissue extracts under identical conditions (limited MS/MS)	> 40 lipid species per neuron (DG granule and CA1 pyramidal neurons)	Not reported	Relative comparisons across neuron groups reported;
Saunders et al. [91]	True single-cell	Nanocapillary sampling of live single cells followed by LC-MS	Analytical flow	Putative IDs reported according to confidence level; only lipid identifications belonging to classes detectable in the internal standards were retained	~ 260 lipids tentatively identified in a single cell	Not reported	Not reported
von Gerichten et al*. *[89]	Both (explicit comparison)	True single cells via capillary sampling of living cells vs bulk extract dilution to cell-equivalent mass	Microflow LC; ~ 15 min method	Coverage counted as lipid features with confirmed MS^2^ (fragmentation-based identification)	60 ± 8 lipids per single PANC-1 cell (n = 11)	“1 cell equivalent” (0.2 cells/µL, 5 µL inj.): 135 ± 2 lipid features	Mean ± 95% CI is referenced for grouped plots; single-cell coverage given with mean ± SD
Kontiza et al. [83]	True single-cell, with blank driven optimization	Microfluidic sorting/dispensing of live single cells into vials; emphasis on contamination control and blank correction	Analytical flow	“Lipid features” after criteria + blank correction; optimized identification increased	After optimization: ~ 210 average lipid features per single cell (blank-corrected; similar pre/post correction)	Not reported	SD/error bars shown; internal-standard RSDs are provided in SI (Table S2)

It is also important to note that the majority of micro-, capillary, and nanoLC-MS lipidomics methods are deployed as global profiling workflows (relative or semi-quantitative) rather than fully absolute quantification platforms. This is not a limitation of miniaturized LC per se, but a consequence of lipidomics physics and logistics: comprehensive absolute quantification would require broad isotopically labeled IS coverage across lipid classes and structural diversity, plus calibration strategies that remain valid under strong matrix effects and class-dependent ionization. Profiling-oriented micro and nanoflow regimes are therefore commonly optimized for coverage, reproducibility, and throughput, reporting comparative abundance changes across conditions rather than absolute cellular concentrations.

A further reason most micro and nanoLC lipidomics remain profiling-first is that truly quantitative workflows demand stable response factors across long batch sequences, not just high sensitivity on a short validation set. In practice, prolonged sequences can introduce and amplify time-dependent drift in retention time, peak shape, carryover, electrospray stability, and matrix deposition at the emitter or inlet, which alters ionization efficiency and effective recovery in ways that are difficult to fully normalize at single-cell or ultra-low input. As a result, many miniaturized LC-MS lipidomics studies prioritize relative quantification with dense QC frameworks (pooled QCs, internal standards, drift correction) rather than claiming broad absolute quantification, because quantitative claims require robustness that does not degrade over hundreds of injections. As a result, quantitative lipidomics at single-cell scale should be interpreted as a hierarchy of achievable accuracy, where relative and semi-quantitative measurements remain robust, while fully absolute quantification is conditional and often very system-specific.

## Complementarity of miniaturized LC-MS with MSI

**Fig. 3:**
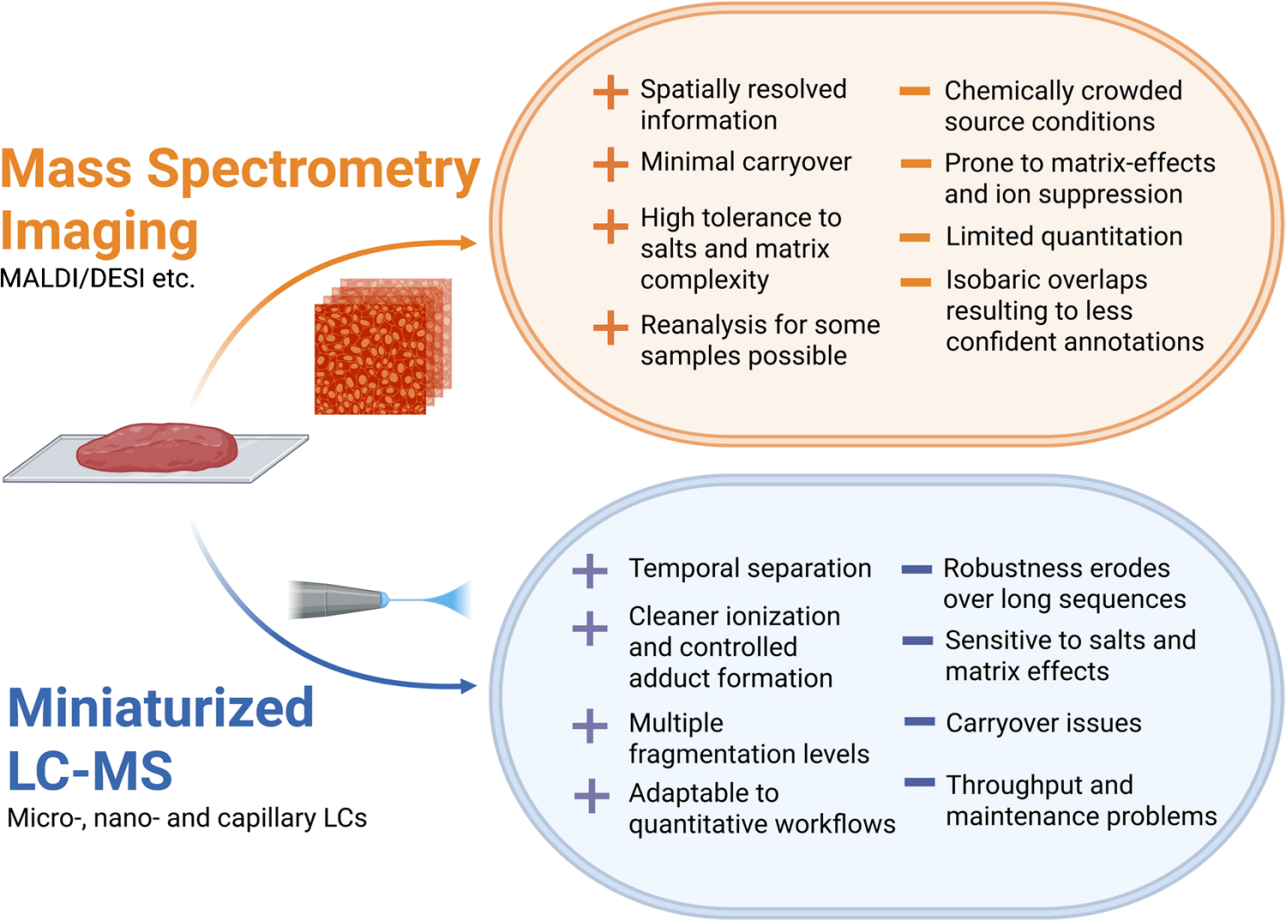
Conceptual comparison of mass spectrometry imaging (MSI) and miniaturized LC–MS for lipid analysis

LC-MS and MSI should be viewed as complementary analytical strategies that operate under different physical constraints, prioritizing molecular specificity and spatial resolution, respectively (Fig. 3). While MSI remains indispensable for visualizing lipid distributions within intact tissues, miniaturized chromatography-MS workflows excel in structural resolution, quantitative accuracy, and detection sensitivity. By combining low-flow separations with advanced ionization and ion mobility strategies, these methods overcome key analytical barriers that constrain imaging-based approaches. The convergence of these complementary technologies is poised to define the next generation of lipidomics, integrating quantitative precision with microscale biological insight.

### Isomeric/isobaric lipid resolution

MSI has transformed spatial lipidomics by enabling direct mapping of molecular distributions from tissue surfaces. Yet, its lack of chromatographic separation limits its ability to resolve isomeric and isobaric lipid species [92, 93]. By contrast, LC coupled to high-resolution MS—especially in miniaturized formats—provides orthogonal separation based on physicochemical properties such as polarity, acyl-chain length, and degree of unsaturation, thereby improving structural specificity [94]. Micro- and nanoLC platforms deliver high peak capacities with minimal sample consumption, allowing discrimination of structural isomers that often co-localize in MSI datasets. For example, LC has resolved positional isomers differing only in the location of a double bond across different lipid classes [95]. At the same time, hydrophilic interaction chromatography (HILIC) efficiently separates lipid classes such as phosphatidylserines and phosphatidylethanolamines [96]. The addition of IMS introduces a further dimension of separation based on gas-phase conformation, enabling isomer discrimination beyond the reach of MSI alone [97]. Together, these multidimensional strategies support more confident lipid identification and quantification from minute samples, forming a robust foundation for high-confidence structural lipidomics at single-cell resolution.

### Minimized unwanted adducts and enhancement of low-abundance species

One of the key analytical benefits of chromatographic separation is its ability to control ion-molecule interactions before ionization. In direct imaging workflows, matrix effects and co-desorption of salts or metabolites frequently produce multiple adduct forms, complicating spectra and reducing quantification accuracy [98, 99]. Chromatography mitigates these effects by temporally resolving analytes from inorganic components and co-extracted contaminants [17, 94]. Low-flow LC further enhances the ionization efficiency of trace lipids by generating smaller, more uniformly charged droplets, resulting in improved desolvation and ion transfer [39, 54]. The reduced droplet size and solvent load also promote more controlled adduct formation, typically favoring protonated and ammoniated ions while suppressing unwanted sodium adducts and water-loss species, which simplifies spectra and improves identification confidence [61]. In a recent multinozzle microflow LC-MS implementation [61], only ~ 6.5% of lipids appeared as multiple adduct forms—compared to ~ 25.9% under conventional analytical-flow ESI, demonstrating a substantial reduction in unwanted adducts and cleaner ion populations, attributed to lower metal ion introduction and faster droplet evaporation that enriches ammonium charge carriers. This is particularly beneficial for detecting rare lipid subclasses, such as oxysterols, phosphoinositide’s, and signaling sphingolipids, which often fall below MSI detection thresholds, as cleaner adduct patterns and higher ionization efficiency together increase sensitivity for low-abundance species. By combining chromatographic retention with optimized nanoelectrospray conditions, miniaturized LC-MS selectively enhances low-abundance species without requiring chemical derivatization or extensive pre-fractionation.

### Reduced ion suppression

Miniaturized LC helps reduce ion suppression compared with MSI, primarily by resolving analytes from coextracted matrix components before ionization, thereby reducing the number of competing species entering the ion source simultaneously. In LC-MS workflows, chromatographic separation spreads compounds across time based on their physicochemical properties, so salts, lipids, and other endogenous molecules do not all co-elute with target analytes. This reduces competition for charge during electrospray and mitigates matrix effects that would otherwise suppress ion formation and reduce signal intensity.

Studies demonstrate that proper chromatographic separation significantly reduces matrix-related ion suppression in electrospray MS by preventing co-elution of analytes with interfering species and by enabling cleanup strategies, such as diverting early-eluting salts to waste before ionization [100]. In contrast, MSI (e.g., MALDI or DESI) desorbs and ionizes all molecular constituents present at a given pixel simultaneously, so abundant or surface-active components commonly suppress the ionization of less abundant analytes, leading to heterogeneous sensitivity and compromised quantification across a tissue section [101]. Additionally, lower nanoflow rates in miniaturized LC produce smaller, more uniformly charged droplets in electrospray, improving desolvation efficiency and further reducing matrix effects compared with the bulkier, more complex ion plumes encountered in MSI ionization.

Together, these features make miniaturized LC-MS inherently more tolerant of complex biological matrices and more effective at detecting low-abundance lipid species than direct imaging approaches without chromatographic separation. Consequently, the choice between LC-MS and MSI is not hierarchical but depends on whether analytical priority is placed on structural resolution, quantitative performance, or spatial context.

## Future perspectives

As miniaturization, automation, and computational integration continue to advance, lipidomics is transitioning from descriptive cataloguing to mechanistic, high-resolution biology. The convergence of chromatographic precision, spatial imaging, and multi-omics integration will empower researchers to dissect lipid metabolism with unprecedented depth and context. These technological and conceptual innovations position miniaturized chromatography-based lipidomics at the forefront of analytical science, driving the field toward comprehensive single-cell and subcellular lipid profiling with direct relevance to systems biology and translational medicine.

Lipid diversity is also a challenge in both data acquisition and analysis. Single, end-to-end workflows combining extraction and detection are limited and often fail to generate a comprehensive lipidomic profile for any given sample [102]. Some lipid classes are also more abundant than others in MS-based experiments, leading to the suppression of other lipids [102]. Additionally, in most samples, the natural abundance of lipid classes should be considered (e.g., membrane versus signaling lipids), as these factors will influence which classes are detected in MS. In traditional lipidomics analysis, the use of “bulk” samples is necessary to minimize variations and heterogeneity [103]. While this results in an averaged snapshot of the global lipidome and the general biochemical state of the sample [103], it is only a portion of the overall picture. At single-cell resolution, the lipidome can be probed in greater detail, unmasking cellular intrinsic heterogeneity and capturing the spatial distribution of relevant biomolecules.

Cell diversity and the heterogeneity of cellular interactions are fundamental questions that single-cell analysis aims to address. Compared to bulk sample lipidomics (and metabolomics), measuring biomolecules at the single-cell level is more challenging and is usually done only when information from larger samples is inadequate. Moreover, when studying disease progression and cell microenvironments, single-cell lipidomics and metabolomics can provide even greater detail than bulk samples, especially in the absence of spatial information on lipid and metabolite distribution.

Another application of single-cell lipidomics and metabolomics would be the analysis of liquid biopsies from circulating tumor cells (CTC), which are potential vectors of cancer metastasis [104]. Single-cell analysis of metabolites is beneficial for CTC detection, as it can provide information about the disease state faster than other methods. Aside from identifying and quantifying metabolites of individual cells, single-cell omics methods can be used for in-depth mechanistic studies, including host-pathogen interactions in infected cells [105, 106] and host-microbiome investigations [107].

Finally, single-cell analysis in the context of spatial information will become very important. Cells do not work in isolation in tissues; they form distinct zones or parts of organs. Several workflows focused on measuring single cells from cell cultures or dissected tissues. Technologies such as laser dissection and microcaption allow the isolation of specific cells from regions of interest in a tissue section. This led to the development of deep visual proteomics, combining spatial information with the advantages of nanoLC-MS/MS [108]. In a similar vein, deep visual lipidomics is expected to emerge and enhance our understanding of lipids and their complex interactions, as illustrated by this study using a laser microdissection-coupled shotgun lipidomics platform that enables quantitative, spatially resolved lipidome analysis from small tissue regions [109]. Similarly, deep lipidome coverage is another goal of miniaturized and single-cell lipidomics, aiming to capture the full structural and quantitative diversity of cellular lipids. In the era of machine learning, big data, and AI-integrated workflows, deep lipidome analysis will enable quantitative mapping of cellular heterogeneity, uncovering lipid-driven regulatory mechanisms and biomarker signatures across developmental and disease states.

## Conclusion and outlook

The continual evolution of lipidomics toward single-cell and subcellular resolution marks a paradigm shift in analytical chemistry and molecular biology. Miniaturized chromatography-based workflows—encompassing micro- and nanoflow LC, capillary, and chip-integrated formats—have redefined what is technically achievable in lipid analysis. These approaches maximize sensitivity and structural resolution while conserving scarce samples, addressing the inherent challenge of quantifying nonamplifiable biomolecules, such as lipids, at cellular and subcellular scales. When coupled with advanced ionization interfaces, ion mobility spectrometry, and high-resolution mass analyzers, miniaturized LC-MS platforms now deliver unparalleled analytical depth and quantitative precision. Though many applications currently are working with single-cell equivalents, e.g., diluted extracts, they pave the way for true single-cell applications. Using single-cell equivalents, analytical systems can be tested for their robustness and reproducibility, which would not be possible with single-cell samples.

However, improvements on the analytical side are only one part. True single-cell application requires also improved sample handling. Methodological innovations in microfluidic extraction, automated liquid handling, and solvent-miniaturized sample preparation have further enabled reproducible lipid analysis from picoliter-scale volumes. This also includes, for example, the addition of IS for accurate quantitation. While in general approaches for quantitation shall not differ between bulk and single-cell lipidomics (e.g., addition of IS as early as possible), the small volumes in true single-cell applications require some rethinking. Currently, no comprehensive workflow for accurate quantification of lipids in single cells exists. However, these will be required for broader insights into lipid biology, e.g., enable calculation of, for example, PE-to-PC ratios for single cells, etc. A major focus of the field needs to be enabling advances in quantification. These advances are complemented by improved data acquisition and standardization strategies, including isotope-labeled internal standards, isotope-labeled cell extracts, and reference materials such as NIST SRM 1950, which ensure quantitative reliability across laboratories. Compared to imaging-based approaches, miniaturized LC-MS provides superior separation of isomeric and isobaric lipids, minimizes adduct formation, and reduces ion suppression, hence, transforming sensitivity into true chemical specificity.

Looking forward, the integration of automation, multiplexed separations, and AI-driven data analysis will accelerate the transition of lipidomics from specialized research to scalable, high-throughput applications. Hybrid MSI-LC platforms and subcellular extraction methods will bridge spatial and structural resolution, enabling mechanistic studies of lipid organization within tissues, organelles, and cellular microenvironments. The next frontier lies in fully integrated multi-omics pipelines, where lipidomic data are contextualized alongside genomic, transcriptomic, and proteomic layers to construct a holistic view of cellular function. Importantly, these advances do not eliminate the fundamental trade-offs between sensitivity, robustness, throughput, and spatial resolution, but instead redefine the operational space in which these parameters can be balanced.

As miniaturization and computation continue to converge, lipidomics is poised to move beyond cataloguing molecular species toward predictive, dynamic modeling of lipid metabolism in health and disease. Miniaturized chromatography-based workflows thus stand not merely as an analytical refinement but as a foundation for the next generation of systems lipidomics—quantitative, spatially resolved, and functionally integrative.

## References

[1] Han X. Lipidomics for studying metabolism. Nat Rev Endocrinol. 2016;12:668–79. 10.1038/nrendo.2016.98. 27469345 10.1038/nrendo.2016.98

[2] Han X, Gross RW. Global analyses of cellular lipidomes directly from crude extracts of biological samples by ESI mass spectrometry: a bridge to lipidomics. J Lipid Res. 2003;44:1071–9. 10.1194/jlr.R300004-JLR200. 12671038 10.1194/jlr.R300004-JLR200

[3] Tharkeshwar AK, Trekker J, Vermeire W, Pauwels J, Sannerud R, Priestman DA, et al. A novel approach to analyze lysosomal dysfunctions through subcellular proteomics and lipidomics: the case of NPC1 deficiency. Sci Rep. 2017(7). 10.1038/srep41408. 10.1038/srep41408 PMC5278418 28134274

[4] Olund Villumsen S, Benfeitas R, Knudsen AD, Gelpi M, Høgh J, Thomsen MT, et al. Integrative lipidomics and metabolomics for system-level understanding of the metabolic syndrome in long-term treated HIV-infected individuals. Front Immunol. 2022;12:742736. 10.3389/FIMMU.2021.742736/BIBTEX. 35095835 10.3389/fimmu.2021.742736 PMC8791652

[5] Casares D, Escribá PV, Rosselló CA. Membrane lipid composition: effect on membrane and organelle structure, function and compartmentalization and therapeutic avenues. Int J Mol Sci. 2019. 10.3390/IJMS20092167. 31052427 10.3390/ijms20092167 PMC6540057

[6] Horn A, Jaiswal JK. Structural and signaling role of lipids in plasma membrane repair. Curr Top Membr. 2019;84:67. 10.1016/BS.CTM.2019.07.001. 31610866 10.1016/bs.ctm.2019.07.001 PMC7182362

[7] El-Fakharany EM, Redwan EM. Protein–lipid complexes: molecular structure, current scenarios and mechanisms of cytotoxicity. RSC Adv. 2019;9:36890–906. 10.1039/C9RA07127J. 35539089 10.1039/c9ra07127j PMC9075609

[8] Van Meer G, De Kroon AIPM. Lipid map of the mammalian cell. J Cell Sci. 2011;124:5–8. 10.1242/JCS.071233. 21172818 10.1242/jcs.071233

[9] Kimura T, Jennings W, Epand RM. Roles of specific lipid species in the cell and their molecular mechanism. Prog Lipid Res. 2016;62:75–92. 10.1016/J.PLIPRES.2016.02.001. 26875545 10.1016/j.plipres.2016.02.001

[10] Hensley CT, Faubert B, Yuan Q, Lev-Cohain N, Jin E, Kim J, et al. Metabolic heterogeneity in human lung tumors. Cell. 2016;164:681–94. 10.1016/j.cell.2015.12.034. 26853473 10.1016/j.cell.2015.12.034 PMC4752889

[11] Lombard-Banek C, Moody SA, Nemes P. Single-cell mass spectrometry for discovery proteomics: quantifying translational cell heterogeneity in the 16-cell frog (*Xenopus*) embryo. Angew Chem Int Ed. 2016;55:2454–8. 10.1002/anie.201510411. 10.1002/anie.201510411 PMC4755155 26756663

[12] Jha D, Blennow K, Zetterberg H, Savas JN, Hanrieder J. Spatial neurolipidomics—MALDI mass spectrometry imaging of lipids in brain pathologies. J Mass Spectrom. 2024;59:e5008. 10.1002/jms.5008. 38445816 10.1002/jms.5008 PMC12013527

[13] Eberlin LS, Liu X, Ferreira CR, Santagata S, Agar NYR, Cooks RG. Desorption electrospray ionization then MALDI mass spectrometry imaging of lipid and protein distributions in single tissue sections. Anal Chem. 2011;83:8366–71. 10.1021/ac202016x. 21975048 10.1021/ac202016x PMC3229813

[14] Vandenbosch M, Mutuku SM, Mantas MJQ, Patterson NH, Hallmark T, Claesen M, et al. Toward omics-scale quantitative mass spectrometry imaging of lipids in brain tissue using a multiclass internal standard mixture. Anal Chem. 2023;95:18719–30. 10.1021/acs.analchem.3c02724. 38079536 10.1021/acs.analchem.3c02724 PMC11372745

[15] Hendriks TFE, Krestensen KK, Mohren R, Vandenbosch M, De Vleeschouwer S, Heeren RMA, et al. MALDI-MSI-LC-MS/MS workflow for single-section single step combined proteomics and quantitative lipidomics. Anal Chem. 2024;96:4266–74. 10.1021/acs.analchem.3c05850. 38469638 10.1021/acs.analchem.3c05850 PMC10938281

[16] Züllig T, Trötzmüller M, Köfeler HC. Lipidomics from sample preparation to data analysis: a primer. Anal Bioanal Chem. 2020;412:2191–209. 10.1007/s00216-019-02241-y. 31820027 10.1007/s00216-019-02241-y PMC7118050

[17] Li M, Yang L, Bai Y, Liu H. Analytical methods in lipidomics and their applications. Anal Chem. 2014;86:161–75. 10.1021/ac403554h. 24215393 10.1021/ac403554h

[18] Tarazona P, Feussner K, Feussner I. An enhanced plant lipidomics method based on multiplexed liquid chromatography–mass spectrometry reveals additional insights into cold- and drought-induced membrane remodeling. Plant J. 2015;84:621–33. 10.1111/TPJ.13013. 26340975 10.1111/tpj.13013

[19] Djambazova KV, Klein DR, Migas LG, Neumann EK, Rivera ES, Van De Plas R, et al. Resolving the complexity of spatial lipidomics using MALDI TIMS imaging mass spectrometry. Anal Chem. 2020;92:13290–7. 10.1021/ACS.ANALCHEM.0C02520/ASSET/IMAGES/LARGE/AC0C02520_0005.JPEG. 32808523 10.1021/acs.analchem.0c02520

[20] Zhang H, Liu Y, Fields L, Shi X, Huang P, Lu H, et al. Single-cell lipidomics enabled by dual-polarity ionization and ion mobility-mass spectrometry imaging. Nat Commun. 2023;2023:1–11. 10.1038/s41467-023-40512-6. 10.1038/s41467-023-40512-6 PMC10457347 37626051

[21] Vasilopoulou CG, Sulek K, Brunner AD, Meitei NS, Schweiger-Hufnagel U, Meyer SW, et al. Trapped ion mobility spectrometry and PASEF enable in-depth lipidomics from minimal sample amounts. Nat Commun. 2020;2020:1–11. 10.1038/s41467-019-14044-x. 10.1038/s41467-019-14044-x PMC6965134 31949144

[22] da Silva KM, Iturrospe E, Heyrman J, Koelmel JP, Cuykx M, Vanhaecke T, et al. Optimization of a liquid chromatography-ion mobility-high resolution mass spectrometry platform for untargeted lipidomics and application to HepaRG cell extracts. Talanta. 2021;235:122808. 10.1016/J.TALANTA.2021.122808. 34517665 10.1016/j.talanta.2021.122808

[23] Li A, Hines KM, Xu L. Lipidomics by HILIC-ion mobility-mass spectrometry. Methods Mol Biol. 2020;2084:119–32. 10.1007/978-1-0716-0030-6_7/COVER. 31729657 10.1007/978-1-0716-0030-6_7 PMC7255642

[24] Chen X, Yin Y, Luo M, Zhou Z, Cai Y, Zhu ZJ. Trapped ion mobility spectrometry-mass spectrometry improves the coverage and accuracy of four-dimensional untargeted lipidomics. Anal Chim Acta. 2022;1210:339886. 10.1016/J.ACA.2022.339886. 35595363 10.1016/j.aca.2022.339886

[25] Rose BS, Leaptrot KL, Harris RA, Sherrod SD, May JC, McLean JA. High confidence shotgun lipidomics using structurally selective ion mobility-mass spectrometry. Methods Mol Biol. 2021;2306:11–37. 10.1007/978-1-0716-1410-5_2/COVER. 33954937 10.1007/978-1-0716-1410-5_2 PMC10127451

[26] Rudt E, Feldhaus M, Margraf CG, Schlehuber S, Schubert A, Heuckeroth S, et al. Comparison of data-dependent acquisition, data-independent acquisition, and parallel reaction monitoring in trapped ion mobility spectrometry-time-of-flight tandem mass spectrometry-based lipidomics. Anal Chem. 2023;95:9488–96. 10.1021/ACS.ANALCHEM.3C00440/ASSET/IMAGES/LARGE/AC3C00440_0005.JPEG. 37307407 10.1021/acs.analchem.3c00440

[27] Paglia G, Smith AJ, Astarita G. Ion mobility mass spectrometry in the omics era: challenges and opportunities for metabolomics and lipidomics. Mass Spectrom Rev. 2022;41:722–65. 10.1002/MAS.21686. 33522625 10.1002/mas.21686

[28] Kotnala A, Anderson DMG, Messinger JD, Curcio CA, Schey KL. Untargeted lipidomic profiling of aged human retina with and without age-related macular degeneration (AMD). Adv Exp Med Biol. 2023;1415:37–42. 10.1007/978-3-031-27681-1_6/COVER. 37440011 10.1007/978-3-031-27681-1_6

[29] Danne-Rasche N, Coman C, Ahrends R. Nano-LC/NSI MS refines lipidomics by enhancing lipid coverage, measurement sensitivity, and linear dynamic range. Anal Chem. 2018;90:8093–101. 10.1021/acs.analchem.8b01275. 29792796 10.1021/acs.analchem.8b01275

[30] Cattaneo A, Martano G, Restuccia U, Tronci L, Bianchi M, Bachi A, et al. Opti-nql: an optimized, versatile and sensitive nano-lc method for ms-based lipidomics analysis. Metabolites. 2021. 10.3390/metabo11110720. 34822378 10.3390/metabo11110720 PMC8623082

[31] Lee JC, Kim YB, Moon MH. Enhancement of acidic lipid analysis by nanoflow ultrahigh performance liquid chromatography–mass spectrometry. Anal Chim Acta. 2021;1166:338573. 10.1016/J.ACA.2021.338573. 34022993 10.1016/j.aca.2021.338573

[32] Harrieder EM, Kretschmer F, Böcker S, Witting M. Current state-of-the-art of separation methods used in LC-MS based metabolomics and lipidomics. J Chromatogr B. 2022;1188:123069. 10.1016/J.JCHROMB.2021.123069. 10.1016/j.jchromb.2021.123069 34879285

[33] Aydoǧan C, Rigano F, Krčmová LK, Chung DS, MacKa M, Mondello L. Miniaturized LC in molecular omics. Anal Chem. 2020;92:11485–97. 10.1021/acs.analchem.0c01436. 32867499 10.1021/acs.analchem.0c01436

[34] Zardini Buzatto A, Kwon BK, Li L. Development of a NanoLC-MS workflow for high-sensitivity global lipidomic analysis. Anal Chim Acta. 2020;1139:88–99. 10.1016/J.ACA.2020.09.001. 33190714 10.1016/j.aca.2020.09.001

[35] Shevchenko A, Simons K. Lipidomics: coming to grips with lipid diversity. Nat Rev Mol Cell Biol. 2010;11:593–8. 10.1038/nrm2934. 20606693 10.1038/nrm2934

[36] Zenobi R. Single-cell metabolomics: analytical and biological perspectives. Science. 2013;342:1243259. 10.1126/science.1243259. 24311695 10.1126/science.1243259

[37] Capolupo L, Khven I, Lederer AR, Mazzeo L, Glousker G, Ho S, et al. Sphingolipids control dermal fibroblast heterogeneity. Science. 2022;376:eabh1623. 10.1126/science.abh1623. 35420948 10.1126/science.abh1623

[38] Dittrich PS, Manz A. Lab-on-a-chip: microfluidics in drug discovery. Nat Rev Drug Discov. 2006;5:210–8. 10.1038/nrd1985. 16518374 10.1038/nrd1985

[39] Wilm M, Mann M. Analytical properties of the nanoelectrospray ion source. Anal Chem. 1996;68:1–8. 10.1021/AC9509519/ASSET/IMAGES/LARGE/AC9509519F00006.JPEG. 8779426 10.1021/ac9509519

[40] Juraschek R, Dülcks T, Karas M. Nanoelectrospray—more than just a minimized-flow electrospray ionization source. J Am Soc Mass Spectrom. 1999;10:300–8. 10.1016/S1044-0305(98)00157-3. 10197351 10.1016/S1044-0305(98)00157-3

[41] Duivenvoorden AAM, Claes BSR, van der Vloet L, Lubbers T, Glunde K, Olde Damink SWM, et al. Lipidomic phenotyping of human small intestinal organoids using matrix-assisted laser desorption/ionization mass spectrometry imaging. Anal Chem. 2023;95:18443–50. 10.1021/acs.analchem.3c03543. 38060464 10.1021/acs.analchem.3c03543 PMC10733903

[42] Yao M, Vaithiyanathan M, Allbritton NL. Analytical techniques for single-cell biochemical assays of lipids. Annu Rev Biomed Eng. 2023;25:281–309. 10.1146/annurev-bioeng-110220-034007. 37068764 10.1146/annurev-bioeng-110220-034007 PMC11032153

[43] Wilson SR, Vehus T, Berg HS, Lundanes E. Nano-LC in proteomics: recent advances and approaches. Bioanalysis. 2015;7:1799–815. 10.4155/bio.15.92. 26270786 10.4155/bio.15.92

[44] Nováková L, Vlčková H. A review of current trends and advances in modern bio-analytical methods: chromatography and sample preparation. Anal Chim Acta. 2009;656:8–35. 10.1016/j.aca.2009.10.004. 19932811 10.1016/j.aca.2009.10.004

[45] Wilm M. Principles of electrospray ionization. Mol Cell Proteomics. 2011;10:M111.009407. 10.1074/MCP.M111.009407. 21742801 10.1074/mcp.M111.009407 PMC3134074

[46] Shan L, Jones BR. Nano-LC: an updated review. Biomed Chromatogr. 2022;36:e5317. 10.1002/bmc.5317. 34981550 10.1002/bmc.5317

[47] Long Z, Zhao Z, Fan X, Luo X. Comparison of analytical-flow, micro-flow and nano-flow LC-MS/MS for sub-proteome analysis. J Pharm Biomed Anal. 2025;252:116484. 10.1016/j.jpba.2024.116484. 39353257 10.1016/j.jpba.2024.116484

[48] Hanson EK, Foster SW, Piccolo C, Grinias JP. Considerations for method development and method translation in capillary liquid chromatography: a tutorial. J Chromatogr Open. 2024;6:100190. 10.1016/j.jcoa.2024.100190. 40092551 10.1016/j.jcoa.2024.100190 PMC11905334

[49] Jorgenson JW. Capillary liquid chromatography at ultrahigh pressures. Annu Rev Anal Chem (Palo Alto Calif). 2010;3:129–50. 10.1146/annurev.anchem.1.031207.113014. 20636037 10.1146/annurev.anchem.1.031207.113014

[50] Xie J, Miao Y, Shih J, Tai Y-C, Lee TD. Microfluidic platform for liquid chromatography−tandem mass spectrometry analyses of complex peptide mixtures. Anal Chem. 2005;77:6947–53. 10.1021/ac0510888. 16255594 10.1021/ac0510888

[51] Fortier M-H, Bonneil E, Goodley P, Thibault P. Integrated microfluidic device for mass spectrometry-based proteomics and its application to biomarker discovery programs. Anal Chem. 2005;77:1631–40. 10.1021/ac048506d. 15762566 10.1021/ac048506d

[52] Rozing G. Micropillar array columns for advancing nanoflow HPLC. Microchem J. 2021;170:106629. 10.1016/j.microc.2021.106629.

[53] Berg HE, Halldórsson S, Bakketeig EA, Thiede B, Sandberg CJ, Lundanes E, et al. Micro‐pillar array columns (µPAC): an efficient tool for comparing tissue and cultured cells of glioblastoma. J Chromatogr Open. 2022;2:100047. 10.1016/j.jcoa.2022.100047.

[54] Keller BO, Sui J, Young AB, Whittal RM. Interferences and contaminants encountered in modern mass spectrometry. Anal Chim Acta. 2008;627:71–81. 10.1016/j.aca.2008.04.043. 18790129 10.1016/j.aca.2008.04.043

[55] Chen S, Zeng J, Zhang Z, Xu B, Zhang B. Recent advancements in nanoelectrospray ionization interface and coupled devices. J Chromatogr Open. 2022;2:100064. 10.1016/j.jcoa.2022.100064.

[56] Kelly RT, Page JS, Zhao R, Qian W-J, Mottaz HM, Tang K, et al. Capillary-based multi nanoelectrospray emitters:improvements in ion transmission efficiency and implementation with capillary reversed-phase LC-ESI-MS. Anal Chem. 2008;80:143–9. 10.1021/ac701647s. 18044958 10.1021/ac701647s PMC2587435

[57] Cech NB, Enke CG. Practical implications of some recent studies in electrospray ionization fundamentals. Mass Spectrom Rev. 2001;20:362–87. 10.1002/mas.10008. 11997944 10.1002/mas.10008

[58] Mortensen DN, Williams ER. Theta-glass capillaries in electrospray ionization: rapid mixing and short droplet lifetimes. Anal Chem. 2014;86:9315–21. 10.1021/ac502545r. 25160559 10.1021/ac502545r PMC4165459

[59] Kim J-S, Knapp DR. Microfabricated PDMS multichannel emitter for electrospray ionization mass spectrometry. J Am Soc Mass Spectrom. 2001;12:463–9. 10.1016/S1044-0305(01)00219-7. 11322193 10.1016/S1044-0305(01)00219-7

[60] Cox JT, Marginean I, Smith RD, Tang K. On the ionization and ion transmission efficiencies of different ESI-MS interfaces. J Am Soc Mass Spectrom. 2015;26:55–62. 10.1007/s13361-014-0998-5. 25267087 10.1007/s13361-014-0998-5 PMC4276539

[61] Girel S, Galmiche M, Fiault M, Mieville V, Nowak-Sliwinska P, Rudaz S, et al. Microflow liquid chromatography coupled to multinozzle electrospray ionization for improved lipidomics coverage of 3D clear cell renal cell carcinoma. Anal Chem. 2025;97:5109–17. 10.1021/acs.analchem.4c06337. 39998250 10.1021/acs.analchem.4c06337 PMC11912133

[62] Hoaglund CS, Valentine SJ, Clemmer DE. An ion trap interface for ESI−Ion mobility experiments. Anal Chem. 1997;69:4156–61. 10.1021/ac970526a.

[63] Meier F, Park MA, Mann M. Trapped ion mobility spectrometry and parallel accumulation-serial fragmentation in proteomics. Mol Cell Proteomics. 2021;20:100138. 10.1016/j.mcpro.2021.100138. 34416385 10.1016/j.mcpro.2021.100138 PMC8453224

[64] Girel S, Meister I, Glauser G, Rudaz S. Hyphenation of microflow chromatography with electrospray ionization mass spectrometry for bioanalytical applications focusing on low molecular weight compounds: a tutorial review. Mass Spectrom Rev. 2025;44:491–512. 10.1002/mas.21898. 38952056 10.1002/mas.21898 PMC11976378

[65] Zheng R, Matzinger M, Mayer RL, Valenta A, Sun X, Mechtler K. A high-sensitivity low-nanoflow LC-MS configuration for high-throughput sample-limited proteomics. Anal Chem. 2023;95:18673–8. 10.1021/acs.analchem.3c03058. 38088903 10.1021/acs.analchem.3c03058 PMC10753523

[66] Coy SL, Krylov EV, Nazarov EG, Fornace AJ, Kidd RD. Differential mobility spectrometry with nanospray ion source as a compact detector for small organics and inorganics. Int J Ion Mob Spectrom. 2013;16:217–27. 10.1007/s12127-013-0135-3. 10.1007/s12127-013-0135-3 PMC3728710 23914140

[67] Zrodnikov Y, Davis CE. The highs and lows of FAIMS: predictions and future trends for high field asymmetric waveform ion mobility spectrometry. J Nanomed Nanotechnol. 2012;3:109e. 10.4172/2157-7439.1000e109. 24163785 10.4172/2157-7439.1000e109 PMC3807102

[68] (2021) Ultrasensitive single-cell proteomics workflow identifies >1000 protein groups per mammalian cell. Chemical Science 12:1001–1006. 10.1039/d0sc03636f 10.1039/d0sc03636f PMC8178986 34163866

[69] Huntley AP, Hollerbach AL, Prabhakaran A, Garimella SVB, Giberson CM, Norheim RV, et al. Development of structures for lossless ion manipulations (SLIM) high charge capacity array of traps. Anal Chem. 2023;95:4446–53. 10.1021/acs.analchem.2c05025. 36820625 10.1021/acs.analchem.2c05025 PMC10634340

[70] Silveira JA, Ridgeway ME, Park MA. High resolution trapped ion mobility spectrometery of peptides. Anal Chem. 2014;86:5624–7. 10.1021/ac501261h. 24862843 10.1021/ac501261h

[71] Kyle JE, Zhang X, Weitz KK, Monroe ME, Ibrahim YM, Moore RJ, et al. Uncovering biologically significant lipid isomers with liquid chromatography, ion mobility spectrometry and mass spectrometry. Analyst. 2016;141:1649–59. 10.1039/C5AN02062J. 26734689 10.1039/c5an02062j PMC4764491

[72] Stow SM, Causon TJ, Zheng X, Kurulugama RT, Mairinger T, May JC, et al. An interlaboratory evaluation of drift tube ion mobility - mass spectrometry collision cross section measurements. Anal Chem. 2017;89:9048–55. 10.1021/acs.analchem.7b01729. 28763190 10.1021/acs.analchem.7b01729 PMC5744684

[73] Leaptrot KL, May JC, Dodds JN, McLean JA. Ion mobility conformational lipid atlas for high confidence lipidomics. Nat Commun. 2019;10:985. 10.1038/s41467-019-08897-5. 30816114 10.1038/s41467-019-08897-5 PMC6395675

[74] Baker ES, Hoang C, Uritboonthai W, Heyman HM, Pratt B, MacCoss M, et al. METLIN-CCS: an ion mobility spectrometry collision cross section database. Nature Methods 2023 20. 2023;12:1836–7. 10.1038/s41592-023-02078-5. 10.1038/s41592-023-02078-5 PMC10843661 37932399

[75] Zhou Z, Tu J, Xiong X, Shen X, Zhu Z-J. LipidCCS: prediction of collision cross-section values for lipids with high precision to support ion mobility-mass spectrometry-based lipidomics. Anal Chem. 2017;89:9559–66. 10.1021/acs.analchem.7b02625. 28764323 10.1021/acs.analchem.7b02625

[76] Rudt E, Feldhaus M, Margraf CG, Schlehuber S, Schubert A, Heuckeroth S, et al. Comparison of Data-Dependent Acquisition, Data-Independent Acquisition, and Parallel Reaction Monitoring in Trapped Ion Mobility Spectrometry-Time-of-Flight Tandem Mass Spectrometry-Based Lipidomics. Anal Chem. 2023;95:9488–96. 10.1021/acs.analchem.3c00440. 37307407 10.1021/acs.analchem.3c00440

[77] Paglia G, Angel P, Williams JP, Richardson K, Olivos HJ, Thompson JW, et al. Ion mobility-derived collision cross section as an additional measure for lipid fingerprinting and identification. Anal Chem. 2015;87:1137–44. 10.1021/ac503715v. 25495617 10.1021/ac503715v PMC4302848

[78] Folch J, Lees M, Stanley GHS. A simple method for the isolation and purification of total lipides from animal tissues. J Biol Chem. 1957;226:497–509. 10.1016/S0021-9258(18)64849-5. 13428781

[79] Bligh EG, Dyer WJ. A rapid method of total lipid extraction and purification. Can J Biochem Physiol. 1959. 10.1139/o59-099. 13671378 10.1139/o59-099

[80] Matyash V, Liebisch G, Kurzchalia TV, Shevchenko A, Schwudke D. Lipid extraction by methyl-tert-butyl ether for high-throughput lipidomics*s⃞. J Lipid Res. 2008;49:1137–46. 10.1194/jlr.D700041-JLR200. 18281723 10.1194/jlr.D700041-JLR200 PMC2311442

[81] Löfgren L, Ståhlman M, Forsberg G-B, Saarinen S, Nilsson R, Hansson GI. The BUME method: a novel automated chloroform-free 96-well total lipid extraction method for blood plasma [S]. J Lipid Res. 2012;53:1690–700. 10.1194/jlr.D023036. 22645248 10.1194/jlr.D023036 PMC3540853

[82] Hyötyläinen T, Orešič M. Optimizing the lipidomics workflow for clinical studies—practical considerations. Anal Bioanal Chem. 2015;407:4973–93. 10.1007/s00216-015-8633-2. 25855150 10.1007/s00216-015-8633-2

[83] Kontiza A, von Gerichten J, Saunders KDG, Spick M, Whetton AD, Newman CF, et al. Single-cell lipidomics: an automated and accessible microfluidic workflow validated by capillary sampling. Anal Chem. 2024;96:17594–601. 10.1021/acs.analchem.4c03435. 39460701 10.1021/acs.analchem.4c03435 PMC11541894

[84] Jia Z, Jiang C, Li J, Belgaid Y, Ge M, Li L, et al. Intelligent single-cell manipulation: LLMs- and object detection-enhanced active-matrix digital microfluidics. Microsyst Nanoeng. 2025;11:133. 10.1038/s41378-025-00962-y. 40624038 10.1038/s41378-025-00962-y PMC12234788

[85] Gadara D, Berka V, Spacil Z. High-throughput microbore LC-MS lipidomics to investigate APOE phenotypes. Anal Chem. 2024;96:59–66. 10.1021/acs.analchem.3c02652. 38113351 10.1021/acs.analchem.3c02652 PMC10782415

[86] He Y, Brademan DR, Hutchins PD, Overmyer KA, Coon JJ. Maximizing MS/MS acquisition for lipidomics using capillary separation and Orbitrap Tribrid mass spectrometer. Anal Chem. 2022;94:3394–9. 10.1021/acs.analchem.1c05552. 35138847 10.1021/acs.analchem.1c05552 PMC8950118

[87] Sorensen MJ, Miller KE, Jorgenson JW, Kennedy RT. Ultrahigh-performance capillary liquid chromatography-mass spectrometry at 35 kpsi for separation of lipids. J Chromatogr A. 2020;1611:460575. 10.1016/j.chroma.2019.460575. 31607445 10.1016/j.chroma.2019.460575 PMC6980658

[88] Merrill CB, Basit A, Armirotti A, Jia Y, Gall CM, Lynch G, et al. Patch clamp-assisted single neuron lipidomics. Sci Rep. 2017;7:5318. 10.1038/s41598-017-05607-3. 28706218 10.1038/s41598-017-05607-3 PMC5509708

[89] von Gerichten J, Saunders KDG, Kontiza A, Newman CF, Mayson G, Beste DJV, et al. Single-cell untargeted lipidomics using liquid chromatography and data-dependent acquisition after live cell selection. Anal Chem. 2024;96:6922–9. 10.1021/acs.analchem.3c05677. 38653330 10.1021/acs.analchem.3c05677 PMC11079853

[90] Lee H, Lerno LA, Choe Y, Chu CS, Gillies LA, Grimm R, et al. Multiple precursor ion scanning of gangliosides and sulfatides with a reverse phase microfluidic chip and quadrupole time of flight mass spectrometry. Anal Chem. 2012;84:5905–12. 10.1021/ac300254d. 22697387 10.1021/ac300254d PMC3402638

[91] Saunders KDG, von Gerichten J, Lewis HM, Gupta P, Spick M, Costa C, et al. Single-cell lipidomics using analytical flow LC-MS characterizes the response to chemotherapy in cultured pancreatic cancer cells. Anal Chem. 2023;95:14727–35. 10.1021/ACS.ANALCHEM.3C02854/ASSET/IMAGES/LARGE/AC3C02854_0003.JPEG. 37725657 10.1021/acs.analchem.3c02854 PMC10551860

[92] Bodzon-Kulakowska A, Suder P. Imaging mass spectrometry: instrumentation, applications, and combination with other visualization techniques. Mass Spectrom Rev. 2016;35:147–69. 10.1002/mas.21468. 25962625 10.1002/mas.21468

[93] Passarelli MK, Ewing AG. Single-cell imaging mass spectrometry. Curr Opin Chem Biol. 2013;17:854–9. 10.1016/j.cbpa.2013.07.017. 23948695 10.1016/j.cbpa.2013.07.017 PMC3823831

[94] Cajka T, Fiehn O. Comprehensive analysis of lipids in biological systems by liquid chromatography-mass spectrometry. Trends Anal Chem. 2014;61:192–206. 10.1016/j.trac.2014.04.017. 10.1016/j.trac.2014.04.017 PMC4187118 25309011

[95] Fabritius M, Yang B. Analysis of triacylglycerol and phospholipid sn-positional isomers by liquid chromatographic and mass spectrometric methodologies. Mass Spectrom Rev. 2026;45:4–36. 10.1002/mas.21853. 37279164 10.1002/mas.21853 PMC12706712

[96] Peterka O, Maccelli A, Jirásko R, Vaňková Z, Idkowiak J, Hrstka R, et al. HILIC/MS quantitation of low-abundant phospholipids and sphingolipids in human plasma and serum: dysregulation in pancreatic cancer. Anal Chim Acta. 2024;1288:342144. 10.1016/j.aca.2023.342144. 38220279 10.1016/j.aca.2023.342144

[97] Paglia G, Kliman M, Claude E, Geromanos S, Astarita G. Applications of ion-mobility mass spectrometry for lipid analysis. Anal Bioanal Chem. 2015;407:4995–5007. 10.1007/s00216-015-8664-8. 25893801 10.1007/s00216-015-8664-8

[98] Liebisch G, Vizcaíno JA, Köfeler H, Trötzmüller M, Griffiths WJ, Schmitz G, et al. Shorthand notation for lipid structures derived from mass spectrometry. J Lipid Res. 2013;54:1523–30. 10.1194/jlr.M033506. 23549332 10.1194/jlr.M033506 PMC3646453

[99] Han X, Gross RW. Shotgun lipidomics: electrospray ionization mass spectrometric analysis and quantitation of cellular lipidomes directly from crude extracts of biological samples. Mass Spectrom Rev. 2005;24:367–412. 10.1002/mas.20023. 15389848 10.1002/mas.20023

[100] Pascoe R, Foley JP, Gusev AI. Reduction in matrix-related signal suppression effects in electrospray ionization mass spectrometry using on-line two-dimensional liquid chromatography. Anal Chem. 2001;73:6014–23. 10.1021/ac0106694. 11791574 10.1021/ac0106694

[101] Dilmetz BA, Lee Y, Condina MR, Briggs M, Young C, Desire CT, et al. Novel technical developments in mass spectrometry imaging in 2020: a mini review. Anal Sci Adv. 2021;2:225–37. 10.1002/ansa.202000176. 38716449 10.1002/ansa.202000176 PMC10989618

[102] Wolf C, Quinn PJ. Lipidomics: practical aspects and applications. Prog Lipid Res. 2008;47:15–36. 10.1016/J.PLIPRES.2007.09.001. 17980916 10.1016/j.plipres.2007.09.001

[103] Wang Z, Cao M, Lam SM, Shui G. Embracing lipidomics at single-cell resolution: promises and pitfalls. TrAC Trends Anal Chem. 2023;160:116973. 10.1016/j.trac.2023.116973.

[104] Lin D, Shen L, Luo M, Zhang K, Li J, Yang Q, et al. Circulating tumor cells: biology and clinical significance. Sig Transduct Target Ther. 2021;6(1):1–24. 10.1038/s41392-021-00817-8. 10.1038/s41392-021-00817-8 PMC8606574 34803167

[105] Ali A, Davidson S, Fraenkel E, Gilmore I, Hankemeier T, Kirwan JA, et al. Single cell metabolism: current and future trends. Metabolomics. 2022;18(10):77. 10.1007/s11306-022-01934-3. 36181583 10.1007/s11306-022-01934-3 PMC10063251

[106] Nguyen TD, Lan Y, Kane SS, Haffner JJ, Liu R, McCall L-I, et al. Single-cell mass spectrometry enables insight into heterogeneity in infectious disease. Anal Chem. 2022;94:10567–72. 10.1021/acs.analchem.2c02279. 35863111 10.1021/acs.analchem.2c02279 PMC10064790

[107] Sharma PV, Thaiss CA. Host-Microbiome Interactions in the era of single-cell biology. Front Cell Infect Microbiol. 2020. 10.3389/fcimb.2020.569070. 33163417 10.3389/fcimb.2020.569070 PMC7591464

[108] Mund A, Coscia F, Kriston A, Hollandi R, Kovács F, Brunner A-D, et al. Deep visual proteomics defines single-cell identity and heterogeneity. Nat Biotechnol. 2022;40:1231–40. 10.1038/s41587-022-01302-5. 35590073 10.1038/s41587-022-01302-5 PMC9371970

[109] Varga-Zsíros V, Migh E, Marton A, Kóta Z, Vizler C, Tiszlavicz L, et al. Development of a laser microdissection-coupled quantitative shotgun lipidomic method to uncover spatial heterogeneity. Cells. 2023;12:428. 10.3390/cells12030428. 36766770 10.3390/cells12030428 PMC9913738

